# Widespread atypical UV-induced mutations form in single-stranded DNA

**DOI:** 10.1126/sciadv.aeg5184

**Published:** 2026-09-11

**Authors:** Cameron Cordero, Brittany N. Vandenberg, Isabelle Mittelstadt, Jordan Murch, Marian F. Laughery, Piotr A. Mieczkowski, John J. Wyrick, Steven A. Roberts

**Affiliations:** ^1^ Department of Microbiology and Molecular Genetics, University of Vermont, Burlington, VT 05405, USA.; ^2^ School of Molecular Biosciences, Washington State University, Pullman, WA 99164, USA.; ^3^ Department of Genetics, Lineberger Comprehensive Cancer Center, University of North Carolina, Chapel Hill, NC 27599, USA.

## Abstract

Persistence of common ultraviolet (UV)–induced lesions, like cyclobutane pyrimidine dimers (CPDs) and pyrimidine-pyrimidone (6-4) photoproducts (6-4-PPs), typically results in C>T substitutions at dipyrimidines: a mutation pattern that composes the single-base substitution (SBS) signature 7 in cancer. Oncogenic melanoma mutations rarely involve SBS7-like substitutions. We recently identified noncanonical UV-induced mutations in yeast that appear to originate from atypical AC and TA photoproducts. While an AC photoproduct could account for formation of BRAF V600K, other melanoma drivers like BRAF V600E and NRAS Q61K involve other mutation types, suggesting possible existence of additional atypical photoproducts. Here, we couple temperature-induced telomeric end resection in yeast with serial UV irradiation and whole-genome sequencing to show UV light induces an extended array of noncanonical mutations in single-stranded DNA (ssDNA). This includes AT>AM, GT>GV, AC>AA, AT>TT, and TA>TT substitutions that are resistant to photo-reversion, indicating that they likely originate from atypical photoproducts. UV-induced mutation spectra in yeast lacking Rad30 indicated that Pol η plays substantial roles in the bypass of CPDs and 6-4-PPs regardless of telomere proximity. Unexpectedly, expression of a mutant DNA pol ε (pol2 M644G) reduced both canonical and noncanonical UV-induced mutations specifically within subtelomeric regions of the genome. This suggests a preferential role for pol ε in the resynthesis of uncapped telomeres, with the M644G mutation conferring accurate lesion bypass capabilities to the replicative polymerase. ssDNA-specific UV lesions provide additional damage-mediated mechanisms for the production of oncogenic mutations in melanoma, such as the BRAF V600E mutation that involves a GT>GA substitution.

## INTRODUCTION

Ultraviolet (UV)–induced mutation signatures have been extensively studied because of the strong link between sun exposure and skin cancer (*1*). The canonical UV mutation signature comprises C>T or CC>TT substitutions in dipyrimidine sequences (i.e., TC, CT, or CC) (*1*–*3*) and results from mutagenic bypass of cyclobutane pyrimidine dimers (CPDs) and pyrimidine-pyrimidone (6-4) photoproducts (6-4-PPs). This UV mutation signature, also classified as single-base substitution (SBS) signature 7 by the Catalog of Somatic Mutations in Cancer (*4*), accounts for nearly 90% of substitutions present in cutaneous melanomas (*5*); however, greater than 50% of melanoma driver mutations do not conform to the canonical UV mutation signature (*6*). For example, the *BRAF* gene, coding for a protein kinase involved in the Ras/mitogen-activated protein kinase signaling pathway important to cellular proliferation (*7*), is the key oncogenic event driving melanomagenesis, being mutated in more than 50% of sequenced primary samples (*8*). BRAF V600K, present in 5 to 10% of melanoma genomes (*9*, *10*), is caused by an AC>TT [modified base(s) underlined] substitution (*7*), whereas BRAF V600E, the most common *BRAF* mutation, results from a GTG>GAG substitution (*11*). Neither mutation involves substitutions in dipyrimidine sequences expected for CPD or 6-4-PP–induced mutations, suggesting that UV exposure may promote these changes through noncanonical mechanisms.

Through whole-genome sequencing (WGS) of serially UV-irradiated yeast, we recently identified several noncanonical UV-induced mutations, including AC>TT and TA>TT substitutions. AC>TT substitutions specifically may contribute to BRAF V600K formation (*12*). Both mutation types shared characteristics of UV-induced mutations, such as exhibiting transcriptional asymmetry and increasing in the absence of the global-genome (GG) nucleotide excision repair (NER) pathway (*12*), indicating that they are likely caused by atypical photoproducts. The A>T substitution likely originates from a thymine-adenine (TA) photoproduct that had been shown to produce this mutation type (*13*–*15*); however, no known AC photoproduct has been described. Reversion mutation assays have shown that UVC light can induce the GTG>GAG BRAF V600E mutation, and similar characterization of UV-induced mutations in Pol η-deficient yeast (*16*) and tumors (*17*) supports the presence of a TG photoproduct, suggesting that other mutagenic atypical UV photoproducts are yet to be identified.

Atypical UV-induced mutations can be difficult to identify and characterize due to the rarity of the lesions. A major constraint on UV photoproduct formation is limited ability to produce the preferable torsional angle and distance between two neighboring bases in double-stranded DNA (dsDNA) (*18*, *19*). Increased flexibility of single-stranded DNA (ssDNA), however, increases formation of most UV dimers (*20*, *21*). Consequently, UV-induced mutations occur 60 times more frequently in ssDNA in yeast (*20*). This higher sensitivity for mutation likely results from the combined effects of increased photoproduct formation and inhibited NER, forcing these lesions to be bypassed during DNA synthesis (*19*, *22*). Additional photoproducts may be ssDNA specific, potentially further augmenting UV mutation in ssDNA.

To identify additional noncanonical mutations associated with atypical photoproducts, we UV irradiated a previously characterized yeast system that generates subtelomeric ssDNA through telomere end resection following temperature-induced inactivation of cdc13-1 (*20*, *23*, *24*). We show that subtelomeric ssDNA generally promotes UV mutagenesis but also expands the types of substitutions observed to include AT>AM, GT>GV, AC>AA, AT>TT, and TA>TT substitutions. The abundance of UV-induced mutations in subtelomeric regions was unaffected by GG-NER deficiency, indicating that the increase in these mutations was primarily due to increased efficiency of lesion formation in ssDNA. In contrast to canonical UV-induced substitutions, identified noncanonical mutation types in subtelomeric regions were largely resistant to CPD and 6-4-PP photolyase treatment supporting atypical photoproducts underlie their formation. Translesion polymerase–based mechanisms dominate bypass of UV-induced mutations as no change in their abundance was observed in yeast lacking homology-directed template switching. Loss of Pol η recapitulated our previous findings (*16*) that Pol η functions primarily in bypass of CPD- and 6-4-PP–like lesions, but not most atypical photoproducts. Unexpectedly, the Pol ε mutant, M644G, lowers the frequency of all UV-induced mutations specifically within subtelomeric regions, suggesting that this polymerase has a major role in the resynthesis of resected telomeres and that the M644G mutation may allow the polymerase to accommodate template lesions during accurate bypass. The increased mutation abundance and diversity of UV-induced substitutions indicate that mutagenesis associated with transiently formed ssDNA regions of the genome could establish important oncogenic mutations during melanogenesis, such as BRAF V600E.

## RESULTS

### UV-induced mutations are enriched in ssDNA

**Fig. 1.**
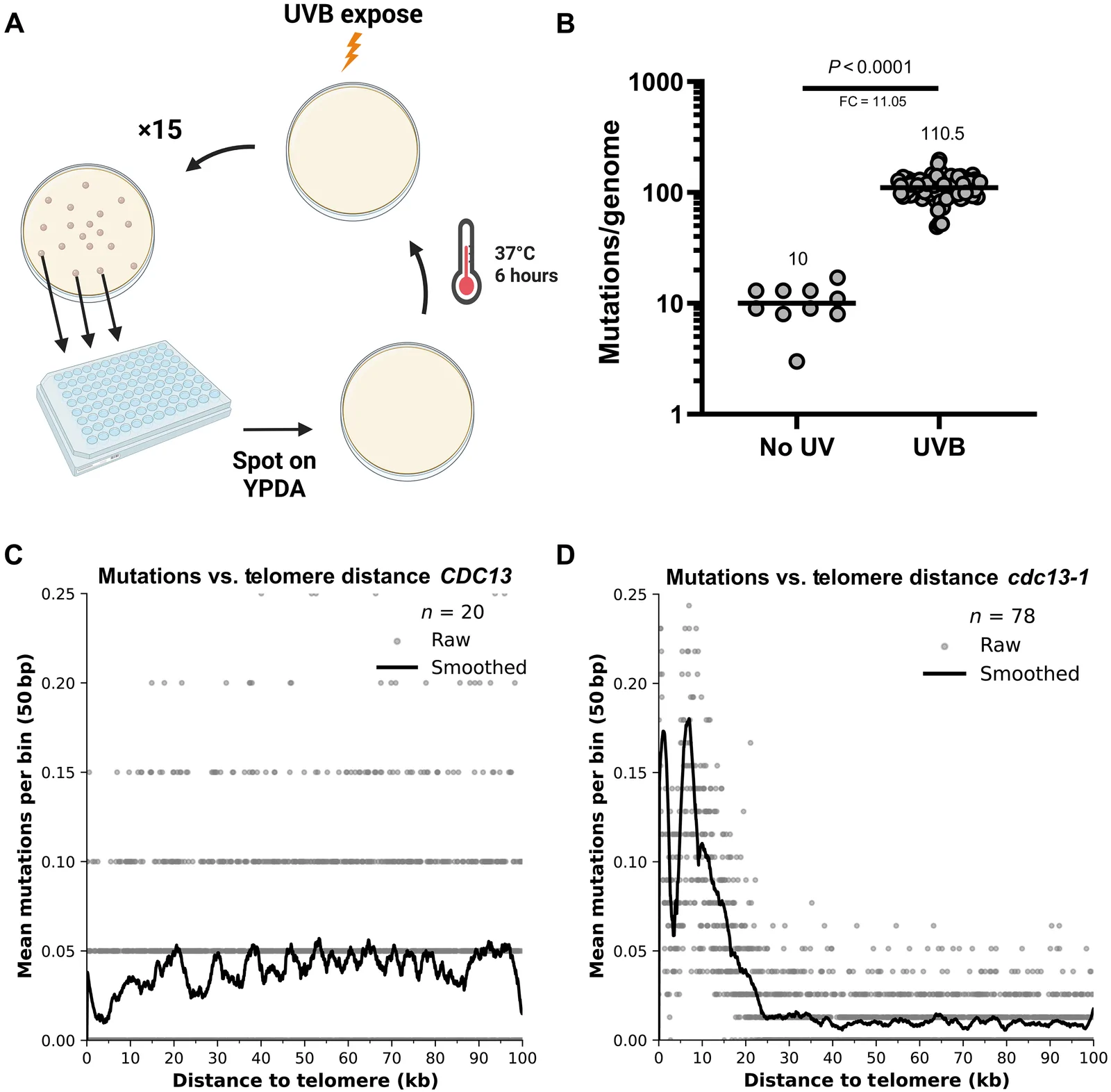
Mutations generated from WGS of UVB-exposed *cdc13*-*1* yeast. (**A**) Experimental procedure to accumulate UV-induced mutations in *cdc13-1* yeast. Independent isolates were resuspended at 10^7^ cells/ml and then spotted onto a YPDA plate. Plates were exposed to a nonpermissive temperature of 37°C for 6 hours and subsequently irradiated with UVB light (150 J/m^2^) and incubated for 3 days. This was repeated 15 times before WGS. Created in BioRender. Vandenberg, B. (2026) https://BioRender.com/e01fw9t. (**B**) Number of mutations per genome sequenced in unexposed (*n* = 10) and UVB-exposed (150 J/m^2^) (*n* = 78) *cdc13-1* yeast. The data were compared by a two-sided Mann-Whitney rank sum test. Median values are shown above the data; *P* value and fold change (FC) are listed above and below the statistical comparison bracket, respectively. (**C**) The number of mutations occurring per 50 bp across the entire genome per sample for UVB-exposed (300 J/m^2^) *CDC13* yeast and (**D**) UVB-exposed (150 J/m^2^) *cdc13-1* yeast, normalized to the total number of samples.

To identify additional atypical UV-induced mutations, we serially UV-irradiated yeast containing resected telomere ends to identify mutations originating from lesions formed in ssDNA. *cdc13-1* yeast was shifted to a nonpermissive temperature of 37°C for 6 hours to allow resection to occur, unexposed or irradiated with UVB (150 J/m^2^), and then allowed to recover. This process was repeated for a total of 15 serial temperature shifts and exposures (Fig. 1A). Forty-three and 10 independent treated and untreated clones, respectively, were then subjected to whole genome sequencing (WGS). Compared with unirradiated cells, UV-exposed *cdc13-1* yeast had an 11-fold elevated substitution density (Fig. 1B), indicating strong global induction of mutations by UV. Previous studies have reported that roughly 30 kb of DNA is resected from telomere ends every 6 hours at nonpermissive temperatures in *cdc13-1* yeast (*23*). Mutation density in the *cdc13-1* strain showed strong enrichment of mutations in the last 20-kb region of the chromosome ends (subtelomeric region), whereas there is no enrichment of mutations in the same regions in *CDC13* yeast (Fig. 1, C and D). Assuming even mutation distribution throughout the genome, we expect roughly 5% of the total mutations to occur in the subtelomeric regions (based on target size) of UV-exposed cells; however, with this system, the subtelomeric region contained more than 35% of all the mutations in the *cdc13-1* yeast, indicating that ssDNA is highly susceptible to UV damage. UV mutation abundance increased steadily with increasing proximity to the telomere ends based on binning mutations within 50–base pair (bp) segments (Fig. 1D). Such a response suggests that the primary determinant of enhanced mutagenesis is the extent of ssDNA formation through resection. Additionally, the lack of specific bins with strongly altered mutagenesis suggests that formation of secondary structures within ssDNA regions that could alter UV lesion formation does not occur with sufficient loci specificity to alter a global mutation distribution analysis.

**Fig. 2.**
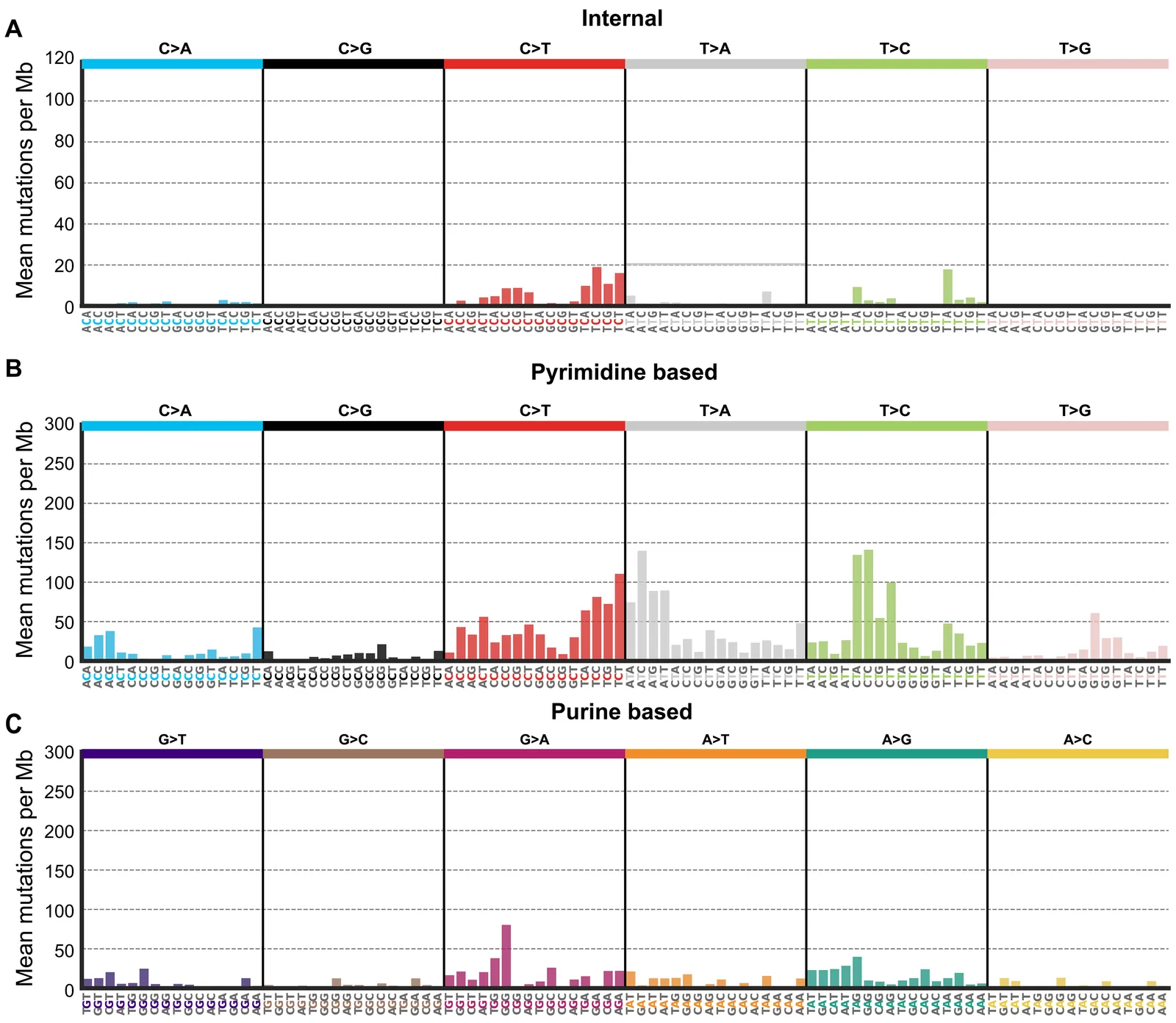
Mutation spectra from WGS of UVB-exposed *cdc13-1* yeast. (**A**) Single mutation profile for UVB-exposed (150 J/m^2^, 15×) *cdc13-1* internal mutations (mutations occurring >20 kb from telomere). Data are presented as mean values of the mutation frequency for each substitution type (e.g., C>A and C>G), and trinucleotide context is depicted. The middle base of each trinucleotide context is mutated. (**B**) Single mutation spectra for pyrimidine-originating mutations occurring in the subtelomeric regions (mutations occurring <20 kb from telomere) in the *cdc13-1* yeast. (**C**) Single mutation spectra for purine-originating mutations occurring in the subtelomeric regions in *cdc13-1* yeast. Mutations shown are from 78 independent isolates exposed to 150 J/m^2^.

5′ to 3′ telomere resection also results in the bottom and top DNA strands remaining, respectively, on the left and right sides of chromosomes (*23*). This characteristic allows the unambiguous determination of the lesion-forming bases rather than needing to infer the originating bases through transcriptional asymmetry analysis, as has been done for UV lesions formed in dsDNA (*12*, *16*). We therefore used this characteristic to define the susceptibility of each possible trinucleotide sequence to UV-induced mutation in ssDNA. We categorized mutations at internal (occurring greater than 20 kb from chromosome ends and predominantly dsDNA) or subtelomeric (occurring within 20 kb from chromosome ends) and normalized each total by the trinucleotide context of the respective regions to account for sequence content differences between the internal and subtelomeric regions. The internal UV-mutation spectra were comparable to the distribution of mutations in the genome-wide spectra from UVB-irradiated *CDC13* yeast (Fig. 2A) and consisted primarily of TC>TT, CC>CT, and TT>TC substitutions. Similar mutation types, likely arising from CPDs (C>T) and 6-4-PPs (T>C), were also observed within 20 kb from the telomere (Fig. 2B). They occurred at higher rates than their reverse complement (G>A and A>G) mutations (Fig. 2C), supporting the unequivocal determination of the pyrimidine-strand containing the lesions. Mutations at noncanonical sequences were also observed within ssDNA. This included low levels of TAN>TTN and TGN>TTN mutations previously reported in WT and *rad30*Δ yeast (*12*, *16*). Despite the low occurrence, TAN>TTN were still enriched ∼2.6-fold in ssDNA over dsDNA, approaching the previously reported fivefold higher quantum yield of TA photoproducts in ssDNA (*25*). ATN>AAN, GTN>GGN, ACN>AAN, and NAT>NGT mutations also were present in larger amounts (Fig. 2, B and C). Sequencing of 38 unirradiated control samples confirmed that these mutation types are UV induced, while a prominent GGG>GAG mutation results from repeated ssDNA generation (fig. S1). The absence of some atypical UV-induced mutation types in the dsDNA spectrum suggests that their cognate lesions form specifically in ssDNA, in contrast to CPDs and 6-4-PPs that occur in both contexts. The trinucleotide sequence contexts for ssDNA-specific mutation types would suggest atypical UV photoproducts of AC, AT, and GT, with different substitution patterns at these dinucleotides indicating either potential additional lesions or differential nucleotide incorporation by lesion bypass polymerases. Notably, some A>G mutations were elevated in tripurine sequences, suggesting the potential for purine-based photoproducts. UV exposure also induces tandem substitutions, which occur when neighboring bases with the same lesion mutate. We previously reported frequent generation of AC>TT tandem substitutions in dsDNA as evidence of an atypical UV photoproduct (*12*). We attempted to evaluate whether ssDNA also elevated the formation of this lesion. AC>TT mutations were observed in internal regions of the genome at a mean frequency of 0.15 mutations per genome (fig. S2 and table S1). This frequency is lower than previous reports (*12*, *16*), likely due to irradiation occurring in a synchronized G_2_ arrested culture, which allows for greater NER as the cells restart and progress through the cell cycle. Within the subtelomeric regions, AC>TT tandem mutations were observed at a very low mean frequency of 0.013 mutations per genome. When compared with the expected mean frequency of 0.006 based on relative target size of the subtelomeric and internal regions, AC>TT mutations appear to be elevated in ssDNA; however, the limited number of events (*n* = 1) prevented statistical evaluation. Insufficient target size likely resulted in low levels of all tandem mutations including more common CC>TT substitutions (mean frequency in internal and subtelomeric regions = 0.31 and 0.1, respectively, with 8 subtelomeric mutations observed). As with the AC>TT, the observed frequency compared with expected suggests that CC>TT tandem mutations are elevated in ssDNA, but the lack of total events limits the use of statistics to effectively evaluate the true effect size. Therefore, we excluded rarer tandem mutations from further analysis.

### Elevated lesion formation causes enhanced mutagenicity of ssDNA

**Fig. 3.**
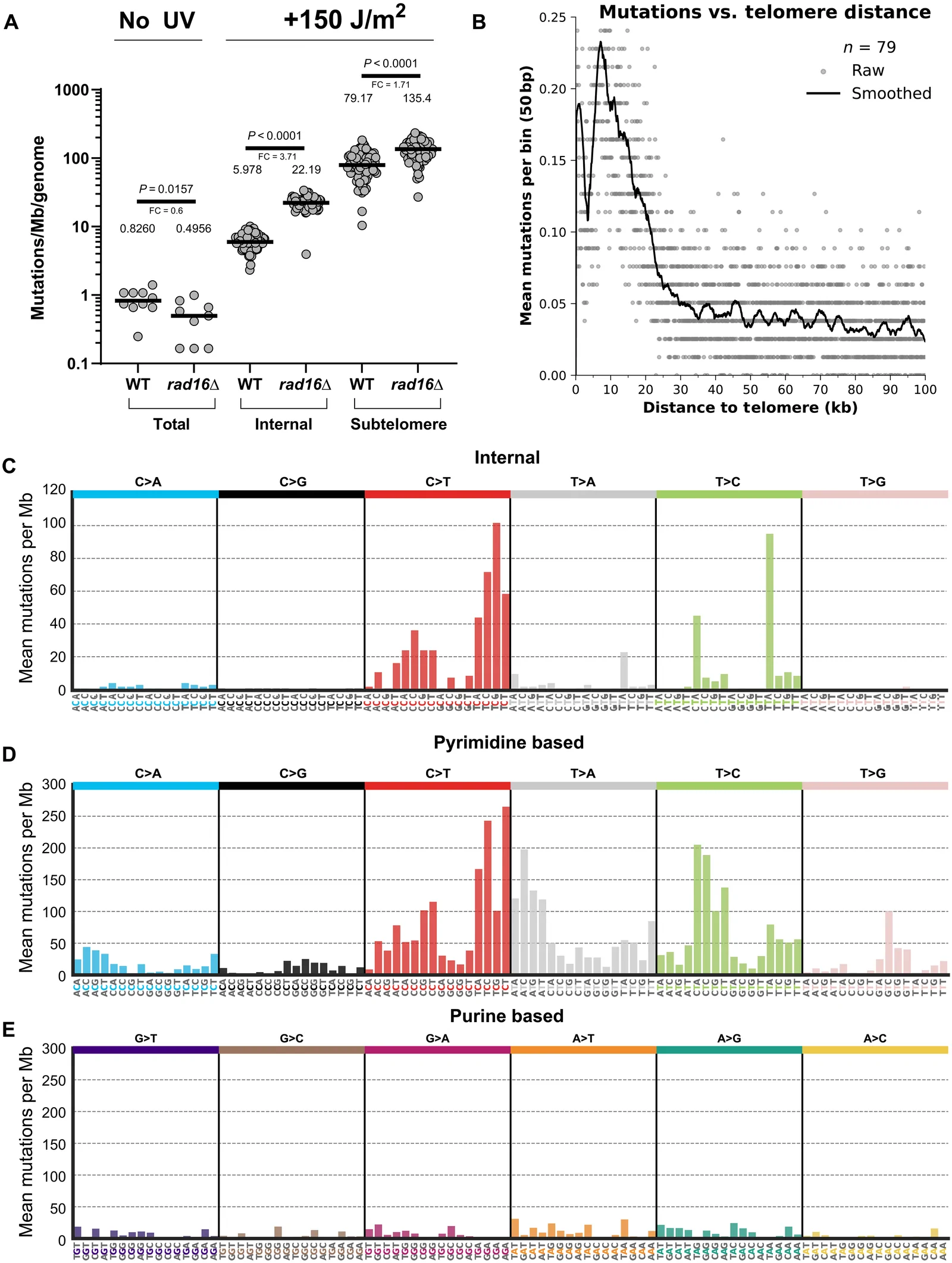
Mutation spectra from WGS of *cdc13-1 rad16*Δ UVB-irradiated yeast. (**A**) Mutations per megabase (Mb) in unexposed (*n* = 9) and UVB-exposed (*n* = 79) (150 J/m^2^) *cdc13-1 rad16*Δ yeast (exposed 15×) in the internal and subtelomeric regions. The median number of mutations was compared by a two-sided Mann-Whitney rank sum test. Median values are shown above the data; *P* value and fold change (FC) are listed above and below the statistical comparison bracket respectively. (**B**) The number of mutations occurring per 50 bp across the entire genome per sample in UVB-exposed *cdc13-1 rad16*Δ yeast. (**C**) Mutation profile for UVB-exposed (150 J/m^2^) *cdc13-1 rad16*Δ yeast for mutations in the internal region, (**D**) pyrimidine-originating mutations in the subtelomeric regions, and (**E**) purine-originating mutations in the subtelomeric regions. Data are presented as mean values of the mutation frequency for each substitution type (e.g., C>A and C>G), and trinucleotide context is depicted. The middle base of each trinucleotide context is mutated.

UV-induced lesions are typically removed from DNA by NER, which requires a template DNA strand to complete repair (*26*). Consequently, enhanced mutagenicity of ssDNA by UV results from either enhanced lesion formation or reduced repair capacity. To determine whether the mutation types observed in the subtelomeric regions form more efficiently in ssDNA or whether they are able to form because NER cannot function on ssDNA, *cdc13-1 rad16*Δ yeast that lack functional GG-NER (*26*) were also subjected to the same protocol before WGS. UVB-irradiated *cdc13-1 rad16*Δ yeast accumulated 56-fold more mutations compared with unexposed *cdc13-1 rad16*Δ yeast (calculated from mean total mutations in each condition) and 3.7-fold more mutations than the UVB-exposed *cdc13-1* yeast within internal dsDNA regions of the genome. Mutations in subtelomeric regions were only 1.6-fold higher in UV exposed *cdc13-1 rad16*Δ yeast compared with *cdc13-1* yeast (Fig. 3A). The relatively small increase in mutations in strains lacking GG-NER suggested that the enrichment of mutations in the subtelomeric ssDNA regions is independent of DNA repair and results primarily from enhanced lesion formation (Fig. 3B). Still, UV-induced subtelomeric mutations were elevated in the *rad16*Δ strains despite ssDNA being a poor substrate for this repair pathway. This increase may be due to persistent lesions that are bypassed in an error-free manner initially but bypassed incorrectly later (*27*), lesions occurring in patches of dsDNA formed during telomere resynthesis where NER could remove the lesion, or lesions formed in unresected subtelomere regions because resection occurs at roughly 15% of chromosome ends upon temperature shifting the *cdc13-1* strains (*28*). The spectra of mutations in both the internal and subtelomeric regions of the genome were largely unaffected by the loss of Rad16 (compare Fig. 2, A to C, to Fig. 3, C to E), consistent with the lack of GG-NER activity in ssDNA regions and previously published data indicating that *rad16*Δ does not affect the composition of UV-induced mutations occurring in dsDNA (*12*). This suggests that the elevated levels of mutations in the subtelomeric regions of the genome are driven predominantly by elevated lesion formation in ssDNA.

### UV dose-dependent formation of photoproducts

**Fig. 4.**
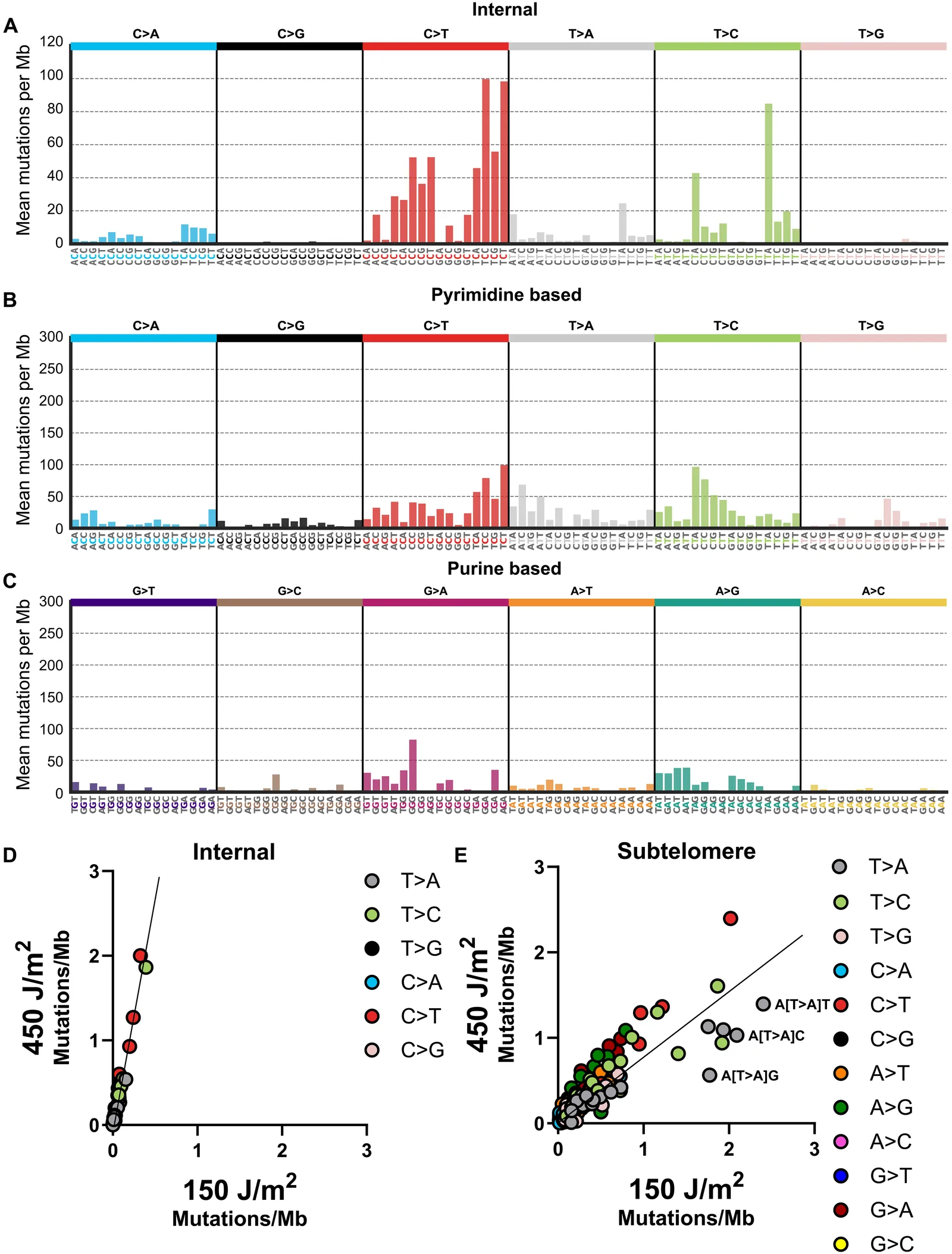
Single mutation spectra *in cdc13-1* yeast irradiated with UVB (450 J/m^2^). (**A**) Single internal mutation profile, (**B**) pyrimidine-originating mutations in the subtelomeric regions, and (**C**) purine-originating mutations in the subtelomeric regions for *cdc13-1* yeast exposed to UVB (450 J/m^2^). Data are presented as mean values of the mutation frequency for each substitution type (e.g., C>A and C>G), and trinucleotide context is depicted. The middle base of each trinucleotide context is mutated. (**D**) Ratio of each mutation type occurring per megabase (Mb) in *cdc13-1* yeast exposed to 150 J/m^2^ (*n* = 78) compared with that in *cdc13-1* yeast exposed to 450 J/m^2^ (*n* = 71) in the internal region and (**E**) subtelomeric region.

We next attempted to increase the number of rarer UV-induced mutations in our dataset by increasing the dose of UV light in our exposure protocol. We exposed *cdc13-1* yeast to UVB (450 J/m^2^) serially and performed WGS on the resulting clones. Consistent with prior work, dsDNA regions showed a proportional increase in mutation density with increasing dose (*12*), indicating that the number of photoproducts formed in dsDNA regions scales with exposure energy. In contrast, ssDNA exhibited no clear increase between 150 and 450 J/m^2^, suggesting that UV-induced damage in ssDNA reaches a mutagenic limit at physiological UVB doses. Both internal and subtelomeric spectra of mutations were nearly identical between doses (compare Fig. 2, A to C, to Fig. 4, A to C), except for ATN>AAN substitutions, which form ∼1.91-fold more readily at lower doses. Canonical UV lesions, like CCN>CTN substitutions, are elevated at 450 J/m^2^, suggesting that atypical UV photoproducts may form ∼1.68-fold more readily at lower dose UV in ssDNA (Fig. 4, D and E).

To evaluate the dose dependence of UV-induced mutation in ssDNA, we measured cell growth and canavanine resistance (CAN-R) established by the mutation of the *CAN1* gene positioned 7.5 kb from the end of chromosome V (*29*) in the presence of the *cdc13*-*1* allele. Compared with *CDC13* strains, end-resected and UV-irradiated *cdc13*-*1* yeast displayed reduced colony formation starting at a UVB dose of 150 J/m^2^ (fig. S3A). This reduction was most evident at a UVB dose of 450 J/m^2^, suggesting that the combination of ssDNA formation and UV damage reduces cell propagation potentially by limiting chromosome end resynthesis. UV-induced mutagenesis at *CAN1* positioned in ssDNA formed at the chromosome V subtelomere also followed a dose dependence, where increased CAN-R could be observed at as little at 5 J/m^2^ and increased until reaching a limit at 45 J/m^2^ (fig. S3B). At greater doses (particularly, UVB at 450 J/m^2^), CAN-R frequency was reduced, likely due to the lower cell propagation at this dose. These results highlight the extreme sensitivity of ssDNA to UV damage where mutagenesis can be observed at doses 30 times lower than typical physiological exposures, whereas, at higher doses, the production of multiple lesions likely reduces end resynthesis, thereby limiting mutation detection.

### Noncanonical mutations arise from atypical photoproducts

**Fig. 5.**
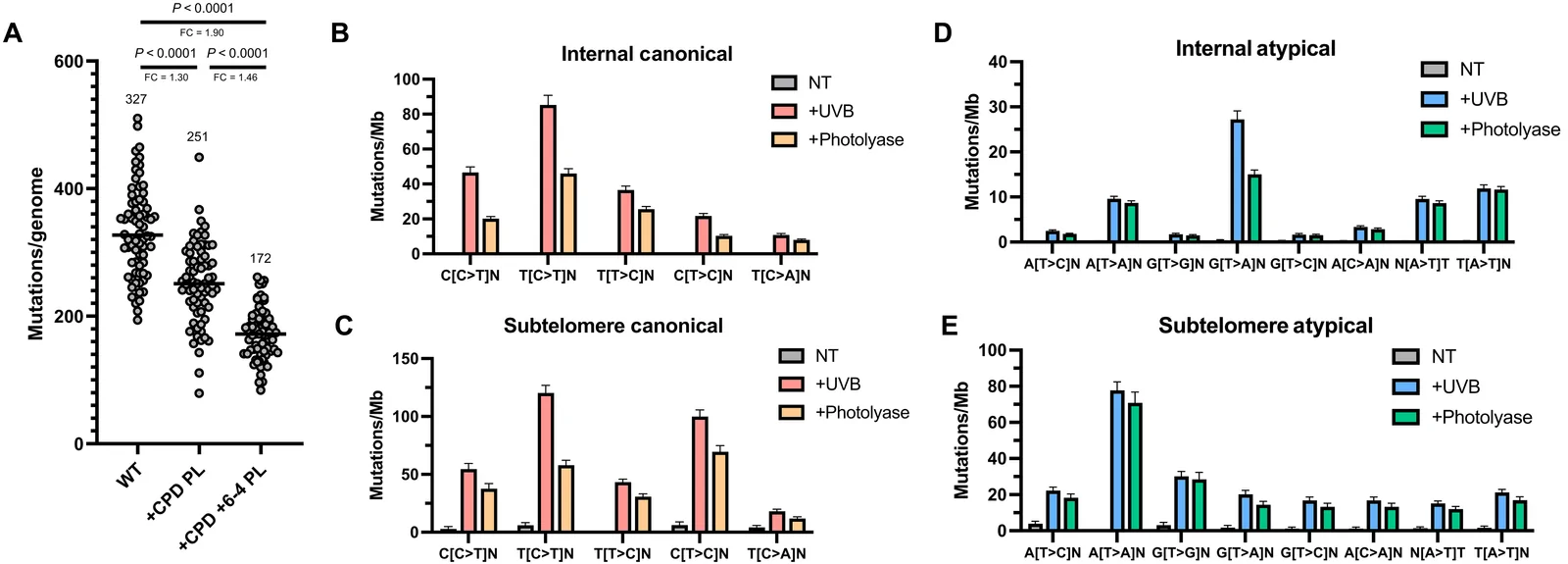
Mutation spectra from WGS of UVB-irradiated cdc13-1 yeast expressing CPD and 6-4 photolyase. (**A**) Mutations per genome sequenced in UVB (450 J/m^2^)–irradiated *cdc13-1* yeast with no photoreactivation (WT, *n* = 71), with photoreactivation to induce endogenous CPD photolyase activity (+CPD PL, *n* = 71), and with photoreactivation in a strain also expressing 6-4 photolyase (+CPD +6-4 PL, *n* = 71). The data were compared by a two-sided Mann-Whitney rank sum test. Median values are shown above the data; *P* value and fold change (FC) are listed above and below the statistical comparison bracket, respectively. (**B**) Mutations arising from canonical UV-induced lesions in the internal and (**C**) subtelomeric regions of UVB-irradiated *cdc13-1* yeast with or without photolyase treatment. (**D**) Mutations arising from atypical UV-induced lesions in the internal and (**E**) subtelomeric regions of UVB-irradiated *cdc13-1* yeast with or without photolyase treatment. Pairwise comparisons were performed using chi-square tests with false discovery rate (FDR) correction; see tables S3 and S4.

To determine whether the extended mutation types observed in ssDNA are a result of atypical photoproducts or canonical CPDs and 6-4-PPs occurring at increased sequences, we assessed the ability of lesions to withstand enzymatic photo-reversion. We integrated the gene encoding *Drosophila* 6-4 photolyase into the genome of *cdc13-1* yeast to complement the endogenous CPD photolyase in the organism. We then repeated our serial exposure protocol with the addition of a photoreactivation step with exposure to UVA immediately following the UVB irradiation to activate both the CPD and 6-4 photolyase enzymes and reduce mutation types associated with canonical UV lesions. Photoreactivation of *cdc13-1* yeast significantly reduced the number of mutations per genome due to the activity of endogenous CPD photolyase (Fig. 5A). The total number of mutations per genome in yeast that also contain 6-4 photolyase was further depleted. C>T and T>C mutations at dipyrimidines were significantly reduced in internal genomic locations, consistent with their likely origin from CPDs and 6-4-PPs (Fig. 5, B and C). Additionally, C>A mutations occurring at dipyrimidines also were reduced by the photolyases, suggesting that these mutations are also caused by these lesions, but through a polymerase alternatively inserting a thymine across the photoproduct instead of the more common adenine insertion. Reduction of these canonical mutation types also occurred in subtelomeric regions, indicating that the photolyases maintain the ability to revert canonical photoproducts in ssDNA. Contrastingly, most of mutations occurring at nondipyrimidine sequences, including AT, GT, AC, and TA dinucleotides, were resistant to photoreactivation in both the internal and subtelomeric regions of DNA (Fig. 5, D and E), supporting that the mutations result from mutagenic bypass of atypical UV photoproducts.

### Altered bypass of lesions in ssDNA

**Fig. 6.**
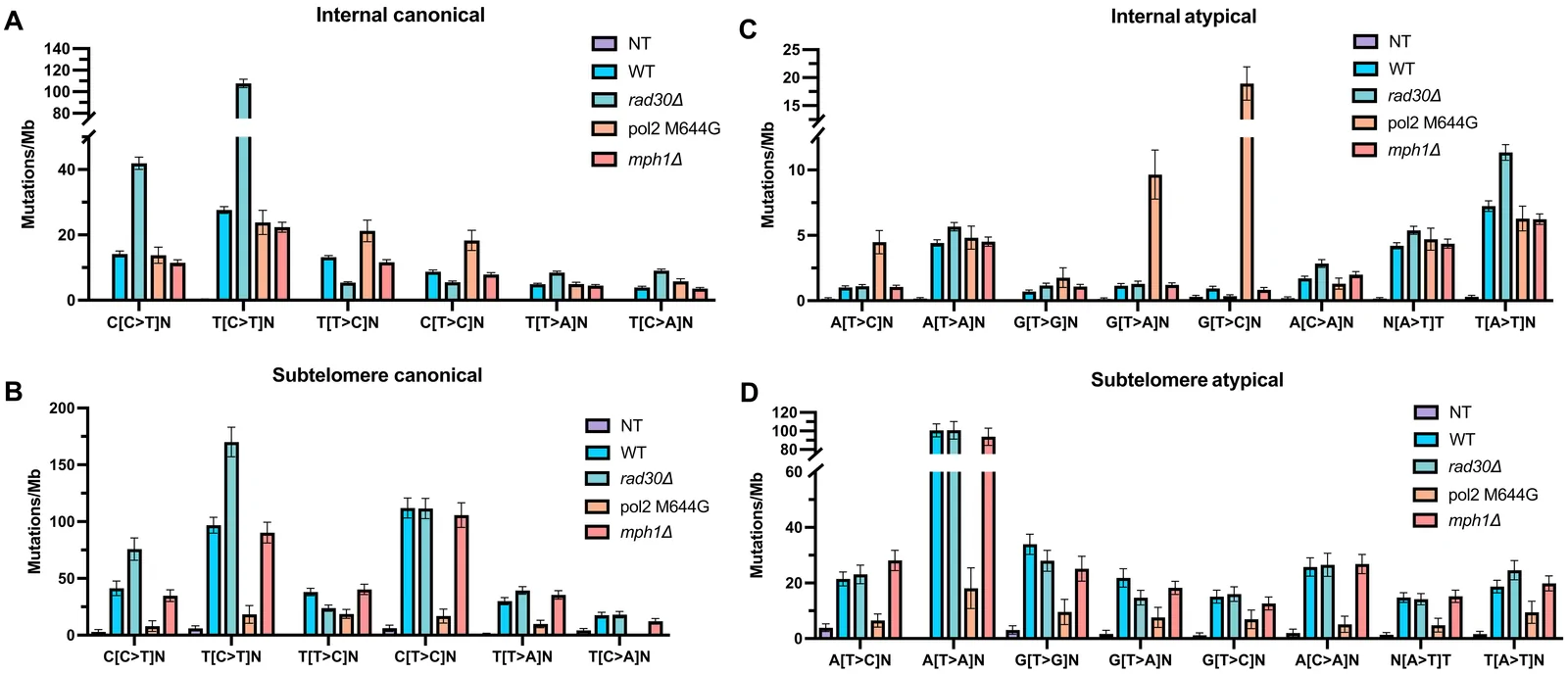
Mutation density within internal and subtelomeric regions in cdc13-1 mutant yeast strains. (**A**) Mutations per megabase (Mb) of mutations arising from canonical lesions occurring in unirradiated *cdc13-1* yeast (NT; *n* = 26), UVB-irradiated *cdc13-1* yeast (WT; *n* = 78), UVB-irradiated *cdc13-1 rad30*Δ yeast (*n* = 57), UVB-irradiated *cdc13-1* pol2 M644G yeast (*n* = 17), and UVB-irradiated *cdc13-1 mph1*Δ yeast (*n* = 63) in the internal region and (**B**) subtelomeric regions. (**C**) Mutations per Mb of mutations arising from atypical lesions occurring in unirradiated *cdc13-1* yeast (NT; *n* = 38), UVB-irradiated *cdc13-1* yeast (WT; *n* = 78), UVB-irradiated *cdc13-1 rad30*Δ yeast (*n* = 57), UVB-irradiated *cdc13-1* pol2 M644G yeast (*n* = 17), and UVB-irradiated *cdc13-1 mph1*Δ yeast (*n* = 63) in the internal and (**D**) subtelomeric regions. Statistical significance was calculated using a chi-squared test from mutation raw counts. All comparisons are listed in tables S5 and S6.

Multiple lesion bypass mechanisms can contribute to the abundance of UV-induced mutations (*26*). To determine which pathways and polymerases function in the bypass of atypical UV photoproducts, we conducted WGS of UVB-irradiated *cdc13-1* yeast with modifications to template switching bypass, DNA Pol η, or DNA Pol ε. Cells may use template switching as an error-free method of bypassing DNA lesions by using the sister chromatid as an undamaged template for replication (*26*). In yeast, this mechanism is reliant on the Mph1 helicase (*30*). The total mutation load significantly increased (compared by Mann-Whitney test, *P* = 0.0323) in the unexposed *cdc13-1 mph1*Δ compared with unexposed *cdc13-1* [wild type (WT)] yeast (fig. S4), likely because yeast lacking *MPH1* have a higher rate of spontaneous mutagenesis (*31*). Despite this increase of spontaneous mutations, the abundance and spectra of UV-induced mutations in *cdc13-1 mph1*Δ yeast are nearly identical to those in UV-exposed *cdc13-1* yeast regardless of internal or subtelomeric positioning (Fig. 6 and fig. S1), indicating that UV-induced lesions are primarily bypassed by TLS with little utilization of template switching for this damage type. This result is consistent with previous analyses, indicating a lack of UV sensitivity in *mph1*Δ yeast (*31*).

DNA Pol η is well established as the TLS polymerase that mediates synthesis across canonical UV-induced lesions (*32*). The fidelity of this process depends on the lesion, while bypass of TT containing CPDs is error free (*33*), and bypass of 6-4-PPs is more mutagenic (*34*). We have previously shown that Pol η completes mutagenic bypass across the proposed AC photoproduct to contribute to AC>TT mutation formation (*16*). To further investigate the involvement of Pol η at rare, atypical UV-induced lesions occurring in ssDNA, we used a previously characterized *cdc13-1 rad30*Δ strain that lacks Pol η (*16*). Induction of telomere end resection and exposure to UVB results in a 2.4-fold increase in total mutation load in the *cdc13-1 rad30*Δ strain compared with the UVB-exposed *cdc13-1* strain (fig. S5). The number of mutations per genome is slightly elevated in the internal region of the *cdc13-1 rad30*Δ strain compared with that of the WT (*cdc13-1*), which is expected on the basis of previously reported data (*16*). The internal mutation spectra for the *cdc13-1 rad30*Δ strain have the same distribution of mutation types as our previously reported UVB-induced spectrum in *rad30*Δ*/rad30*Δ yeast (fig. S5) (*16*). C>T mutations increased compared with those in WT cells, as expected due to the error-free nature of bypass by Pol η in WT cells (Fig. 6A). However, T>C mutations decrease in the *rad30*Δ yeast, indicating that Pol η is mutagenic at the lesions responsible for this mutation type (Fig. 6A). C>A mutations in a TCN context are also elevated in the *cdc13-1 rad30*Δ strain, which is consistent with our previous findings that these mutations likely originate from CPD lesions (Fig. 5B). These observations do not completely extend to the subtelomeric region where lesions form in ssDNA. C>T mutations are still elevated in the *cdc13-1 rad30*Δ strain compared with WT, but, to a lesser extent, CCN mutations increase by 1.8-fold compared with 2.3-fold internally and TCN mutations increase by 1.7-fold compared with 3.3-fold internally (Fig. 6, A and B), suggesting that Pol η bypass is occurring less frequently in this region. TTN>TCN mutations decrease by 1.6-fold in the subtelomeric region in the absence of Pol η, although not to the same extent as seen internally (2.4-fold). Neither CTN>CCN mutations nor C>A mutations changed (Fig. 6B). Atypical mutation types that were not previously seen to be affected by the loss of Pol η, like TAN>TTN mutations, are slightly elevated in the internal and subtelomeric regions of the *cdc13-1 rad30*Δ yeast (Fig. 6, C and D).

We have also previously implicated replicative Pol ε in the bypass of atypical UV-induced lesions using a single-stranded mutation reporter system in a *cdc13-1* pol2 M644G strain, which decreased the number of A>T and AC>TT mutations in the UV-irradiated yeast (*16*). To examine whether this bypass ability extended to the entire genome, we subjected this strain to the WGS protocol detailed above. Mutagenesis is increased in the internal dsDNA regions of the genome for this mutant (fig. S6), with T>C and T>A mutations particularly elevated (Fig. 6, A and C). These substitution patterns are in alignment with previous characterizations of the spontaneous mutagenic effects of pol2 M644G (*35*, *36*), suggesting that they likely accumulated during replication of undamaged DNA during the serial exposure/outgrowth process. In the subtelomeric region, however, this mutant has an opposing effect. The subtelomeric spectrum reveals that this mutant diminishes mutagenesis across all mutation classes in the UV-irradiated subtelomeric region (fig. S6), consistent with our prior reversion reporter results. The specificity of reduced mutation in the subtelomeric regions suggests that Pol ε may play an important role in the resynthesis of subtelomeric DNA following end resection. The reduction of subtelomeric mutations in pol2 M644G strains could result from reduced ability to resynthesize the UV damaged resected chromosome end, thereby selecting for cells with lower lesion abundance, enhanced chromosome end loss, or enhanced bypass fidelity with the M644G mutation. To distinguish between these possibilities, we assessed pol2 M644G growth following end resection and UVB irradiation. Yeast with this mutation displayed similar growth as WT strains, suggesting that they complete resynthesis of the subtelomere and release from the G_2_ arrest over a near WT timeframe (fig. S7A). Moreover, sequenced pol2 M644G outgrowth clones maintained chromosome ends similarly to WT (fig. S7B). Combined, these data suggest the M644G mutation makes the polymerase more accurate in the bypass of UV lesions during this process, potentially due to increased flexibility of the active site allowing the polymerase to more readily accommodate a photodimer within the template strand.

## DISCUSSION

ssDNA is more susceptible to UV-induced damage than dsDNA (*20*, *21*, *24*, *37*). The absence of duplex constraints exposes individual bases to the solvent and allows greater conformational flexibility, which increases the probability that adjacent nucleotides transiently adopt the torsional angles and distances required for covalent bond formation (*18*). This conformational relaxation expands the range of photochemical reactions that can occur, permitting formation of additional types of photoproducts that are sterically restricted in the rigid geometry of B-form dsDNA (*38*). Additionally, ssDNA can form secondary structures (e.g., hairpins and G-quadruplexes) whose three-dimensional conformations may promote certain UV photoproducts at positions with strong strand bends while limiting lesions at regions of dsDNA character. The overall impact of structures formed in ssDNA on UV-induced mutagenesis remains to be determined; however, the increased confirmational flexibility of ssDNA likely increases photochemical reactivity and underlies the strong enrichment of mutations that we observed within resected subtelomeric regions. These mutations arise largely independently of NER, indicating that elevated lesion formation rather than defective DNA repair drives the mutational burden in ssDNA regions. Increased sensitivity in ssDNA may also allow lower doses more in line with physiological UV exposures to generate lesions.

The expanded mutation spectrum in ssDNA regions includes multiple classes that persist even after photoreactivation, suggesting the presence of previously unidentified photolyase-resistant lesions. They occur across a wider range of dinucleotide contexts such as TA, AC, AT, and GT, indicating that more sequence combinations are photochemically reactive in ssDNA. Although the structural identities of the causative lesions remain undefined, the tendency of similar UV-induced SBSs to group within specific neighboring nucleotide sequences suggests that the causative lesions are dinucleotide cross-link photoproducts. Moreover, prior biophysical work shows that base stacking geometry and torsional alignment dictate lesion formation rates in dsDNA (*38*), providing a plausible mechanism by which structural relaxation in ssDNA enables enhanced ability to form such atypical cross-links. However, UV light can promote additional mutagenic lesions, including nonadjacent cross-links (*39*), oxidative base damage through the production of reactive oxygen species (ROS) (*40*), and cytidine deamination induced by photohydration (*41*). Contribution of these damages at some level to the ssDNA UV-induced mutation spectrum cannot be excluded. ROS-induced 8-oxo-guanine, for example, could produce some of the G>T substitutions observed (Fig. 2), although they occur at a different set of trinucleotide sequences than experimentally induced 8-oxo-guanine (*42*). Similarly, cytidine photohydration and deamination, which preferentially occurs in ssDNA, could contribute some of the C>T substitutions. This process, however, would likely contribute a minority of these mutations as photo-reversion significantly reduced C>T mutations (Fig. 5).

The canonical bypass mechanisms of these lesions remain uncertain because telomeric resynthesis likely differs from standard replication, potentially altering individual polymerase usage. Deletion of Pol η increased C>T substitutions while reducing T>C, consistent with its known role in accurate bypass of CPDs (*43**,* *44*) and more error-prone bypass of 6-4-PPs (*34*). These effects were diminished in subtelomeric regions, suggesting that Pol η is less engaged at resected chromosome ends or during postresection resynthesis. In contrast, the pol2 M644G mutant exhibited a substantial reduction in both canonical and atypical substitutions, specifically within subtelomeric DNA, implying that Pol ε plays the dominant role in telomere resynthesis. After telomere uncapping, extensive 5′ to 3′ resection generates long ssDNA tracts that must be resynthesized before replication can resume. Pol ε likely performs much of this resynthesis. The lack of a detectable growth defect or chromosome loss when pol2 M644G yeast are subjected to end resection and subsequent UV exposure (fig. S7) suggests that pol2 M644G may be sufficient to bypass lesions in a relatively error-free manner instead of relying on TLS polymerases. Additional biochemical characterization of the UV lesion bypass fidelity of pol2 M644G is required to confirm this possibility. Some lesions are likely processed accurately, whereas others persist unrepaired for prolonged periods and are eventually bypassed in an error prone manner, similar to lesions that persist in DNA for extended periods in other systems (*27*, *45*). The differences in mutagenic bypass of photoproducts during telomere resynthesis compared with replication-associated bypass may help to explain why the enrichment of some UV-induced mutations in ssDNA falls short of the expected induction based on quantum yield measurements (e.g., TA>TT mutations are enriched 2.6-fold, but TA photoproducts are enriched 5-fold). Increased usage of polymerases that more accurately bypass lesions during telomere resynthesis, usage of an Mph1-independent template switching mechanism of bypass, or potential repair of some lesions through transient annealing of complementary DNAs could all lower the observed mutation enrichment in these regions. How UV photoproducts formed in ssDNA are bypassed in human cells may differ in some aspects compared with that in yeast. Human cells encode more TLS polymerases than yeast (*46*) that could be used in specialized situations like ssDNA gap filling. These additional polymerases may alter the specific mutagenicity and spectrum of ssDNA-associated UV lesions.

Transiently ssDNA regions of the genome naturally arise during DNA replication, transcription, recombination, or as repair intermediates (*47*, *48*). In replication, lagging-strand templates (*49*) and stalled polymerases on either strand expose extended ssDNA tracts (*50*), while transcription generates R-loops containing ssDNA on the nontranscribed strand (*51*). These vulnerable intermediates, therefore, represent focal points for noncanonical UV photoproducts and are likely to sustain more lesions per nucleotide than regions in a dsDNA state. However, the effective target size of ssDNA regions compared with that of more persistent dsDNA states will be less, likely leading to the lower abundance of ssDNA-associated mutation types in melanoma genomes. This disparity in mutation abundance will make using strand biases (*52*) to evaluate the potential impact of ssDNA mutagenesis on melanoma genomes difficult as ssDNA-associated mutations will likely be obscured by dsDNA-associated mutations. The source of ssDNA where lesions accumulate could also complicate strand asymmetry analyses. Preferential lesion formation on the nontranscribed strand in these regions may overlap with or amplify the strand asymmetry typically attributed to TC-NER, making the two processes difficult to distinguish but potentially synergistic contributors to strand-biased mutational landscapes seen in melanoma.

The cancer relevance of our findings becomes clear when considering how ssDNA-specific lesions reshape the mutational spectrum. The single-stranded state generates a distinct set of substitution classes, including elevated canonical C>T, C>A, and T>C while also including T>A, T>G, and A>T changes at comparable frequencies, expanding the potential sequence-context specificity of UV-induced mutations. Notably, one of the most notable features is a 10-fold increase in GTG>GAG substitutions (Fig. 4, D and E), the same context as the oncogenic BRAF V600E mutation that dominates human melanomas (*53*). Prior analyses indicated that, while more common, BRAF V600E is enriched in melanomas compared with that in other cancer types to a lesser extent than BRAF V600K (*12*). This phenomenon likely results from the complexity of mutations required to generate each mutation as the GTG-to-AAG mutation required to generate the V600K variant requires a complex mutational event, whereas the GTG to GAG underlying V600E is a simple SBS. Consequently, BRAF V600E is observed in a variety of non-skin cancers [e.g., thyroid and colorectal; (*54*)] due to the existence of multiple mutagenic mechanisms capable of producing this change. Within melanomas specifically, BRAF V600E likely originates from multiple processes with UV-induced mutagenesis of ssDNA regions providing additional fuel for this oncogenic mutation. Our results show that ssDNA-specific photoproducts form efficiently at GT dinucleotides, potentially at rates similar to canonical TCN>TTN mutations in dsDNA. The high rate of lesion formation in ssDNA suggests that the primary constraint on the abundance of some noncanonical UV-induced mutations is the availability of single-stranded intermediates in the genome. Together, these observations support a model in which transient ssDNA regions arising during replication, transcription, and DNA repair are sensitized substrates for atypical UV photochemistry, enabling the formation of oncogenic substitutions that cannot be explained by standard CPD or 6-4-PP chemistry.

## METHODS

### Yeast strains

All strains are descendants of CG379 (*55*) and are listed in table S2. The *cdc13-1* strains were constructed by mating yBV43/yBV44 (*16*), which contain the *cdc13-1* allele, with yML386.2. Spores were selected that contained the *cdc13-1* allele. The GG-NER mutant was constructed in the resulting strain with deletion cassettes. *RAD16* deletions strains (yCAM003 and yCAM004) were generated by amplifying plasmid pJW25 with primers oAH152 (TTTAGATACTCTTGGCCGTATAACTGGTGTACCAACTGAAAAATCagattgtactgagagtgcac) and oAH153 (TCACTTAAAAACTCCGAGAATAATATATAATAAGAGAATAAAATActgtgcggtatttcacaccg) to generate a *rad16::LEU2* cassette. yBV285 and yBV286, respectively, were transformed with the *rad16::LEU2* cassette and deletion strains selected on SC-leu medium. yCAM003 and yCAM004 were polymerase chain reaction (PCR) validated using oTM046 (TGAAGCCTCCGTTATCCCAGGTTCCT) and oAJB089 (AGGGAAACAGTGGGGACTTCT). *MPH1* deletion strains (yCAM001 and yCAM002) were generated by creating a *mph1::G418-R* cassette by amplifying the *mph1* locus of yJH-003 using oTM-1212 (CTGGTAAAACAAGATTGCTTTCATT) and oTM-1215 (TTACTTTTCGGAGTGTAGGAAATTG) primers, which was subsequently transformed into yBV285 and yBV286. yCAM001 and yCAM002 were PCR validated using oTM048 (GGATGTATGGGCTAAATG) and oAJB101 (TGTTACCCTTTTCCCGCC) Strains expressing *Drosophila* 6-4 photolyase, yIM002 and yIM014, were generated by transforming *cdc13-1* strains (yBV285 and yBV286, respectively) with pML157 (*56*), resulting in integration of *Drosophila* 6-4 photolyase gene at *LEU2*. yIM002 and yIM014 were PCR validated using oML333 (CGTTTCGCTCATTGGACA) and oML334 (GTACTGAAGAGGAGGTCGA).

### UV exposure of ssDNA

Yeast cultures were grown overnight to late log phase in YPDA medium. Cells were collected by centrifugation and resuspended in sterile water to less than 10^7^ cells/ml. Aliquots (3 μl) were spotted onto a YPDA plate. Plates were placed at 37°C for 6 hours to induce resection and then immediately exposed to predominantly UVB radiation at 0, 150, or 450 J/m^2^ using an Analytik Jena UVP Crosslinker, CL-3000 M. Plates were then placed in a dark incubator at 23°C for 3 days. Cells were taken from each spot on the plate and resuspended in sterile water to less than 10^7^ cells/ml, then spotted onto a new YPDA plate, incubated at 37°C for 6 hours, and exposed to the same respective dose of UVB. Photoreactivated groups were additionally exposed to UVA using a Spectroline EA-160 for 30 min at 23°C immediately following resection and then incubated. Exposures and ssDNA generation cycles were repeated for all conditions a total of 15 times.

### Yeast growth assays

Populations of wild-type (yBV285 and yBV286) and pol2 M644G–expressing (yBV378 and yBV379) *cdc13*-*1* strains were grown up in 5 ml of liquid YPDA cultures for 16 hours at 23°C. Cells were then resuspended in water at a concentration of 10^6^ cells/ml and diluted 10-fold to a concentration of 100 cells/ml. Each of the seven dilutions for each strain were spotted onto YPDA using 2-μl volumes and then incubated for 6 hours at 37°C to allow for telomere resection. Test plates were irradiated with UVB at 150 J/m^2^ and then returned to 23°C along with the control plates where they were incubated for 96 hours. The plates were imaged using epi white settings on the Bio-Rad ChemiDoc MP imager.

### CAN-R assay

To assess the dose dependence of UVB-induced mutations in ssDNA, we used *cdc13*-*1* (ySR127) and *CDC13* proficient (ySR583) strains containing the *CAN1* gene in the subtelomere chromosome V. Six clonal populations of each strain were grown in 5 ml of liquid YPDA for 16 hours at 23°C and resuspended cells in water at a concentration of ∼10^7^ cells/ml. Resuspended cultures were plated on synthetic complete medium (100 μl of 1:10^4^ dilution of each resuspension) and synthetic arginine dropout medium supplemented with 0.0006% canavanine (100 μl of undiluted resuspension). The plates were incubated for 6 hours at 37°C to allow for telomere resection to occur in *cdc13*-*1* cells and then exposed to varying doses of UVB (0, 1, 5, 15, 45, 150, and 450 J/m^2^). Plates were lastly incubated for 72 hours at 23°C, complete and revertant colonies were counted, and CAN-R frequencies were calculated.

### Whole-genome sequencing

After being exposed and passaged 15 times, fresh single isolates were taken and grown overnight in YPDA medium. Cells were harvested by centrifugation and resuspended first in TE buffer containing β-mercaptoethanol and then in a buffer containing zymolyase [1 M sorbitol, 10 mM Pipes (pH 6.5), and 20 U of Zymolyase] to disrupt the cell wall. Cells were then lysed in TE buffer with 2% SDS. DNA was precipitated with 5 M KOAc and 100% ethanol, washed with 70% ethanol, and resuspended in low EDTA TE buffer [10 mM tris and 1 mM EDTA (pH 7.5 to 8.0)]. A secondary purification was done by vortexing in tris-buffered phenol:chloroform:isoamyl alcohol. The aqueous phase was removed and precipitated with 100% ethanol. Pellets were washed with 70% ethanol and resuspended in 10 mM tris (pH 8.0). The Illumina sequencing library was prepared using a Kapa Hyper preparation kit (Roche, catalog no. KR0961) and sequenced on an Illumina NovoSeq 6000 platform or DNBSEQ-T7 as in (*12*, *16*).

### Mutation calling

Sequencing reads were aligned to the appropriate reference genome using the Burrows-Wheeler Aligner (BWA) **mem** algorithm (*57*) with default parameters. The WT strains were aligned to the SacCer3 genome (NCBI accession GCA_000146045.2) and *cdc13-1* strains were aligned to ySR127 (NCBI accessions CP011547.1, CP011548.1, CP011549.1, CP011550.1, CP011551.1, CP011552.1, CP011553.1, CP011554.1, CP011555.1, CP011556.1, CP011557.1, CP011558.1, CP011559.1, CP011560.1, CP011561.1, CP011562.1, and CP011563.1), a custom reference genome built from the original SacCer3 genome to incorporate engineered rearrangements (*29*). The resulting Sequence Alignment/Map (SAM) files, containing aligned sequence reads, underwent compression into Binary Alignment/Map (BAM) format via the utilization of **samtools view** [SAMtools v1.13 (*58*) using htslib 1.13+ds (*59*)]. Note: All SAMtools steps were executed with default parameters to ensure a standardized approach. Following compression, the BAM files were sorted according to genomic coordinates using **samtools sort** in preparation for eliminating duplicate reads that may result from PCR amplification artifacts during sequencing. These were eliminated using **samtools rmdup** to avoid any influence on subsequent variant calling and analysis. The conversion of the final BAM files to MPILEUP files was carried out using **samtools mpileup**. The final BAM and MPILEUP files were used for the purpose of detecting mutations using multiple mutation callers.

The BAM files underwent processing using Strelka (v2.9.10) (*60*) and Somatic Sniper (v1.0.5.0) (*61*), whereas the MPILEUP files were processed using VarScan (v2.3) (*62*). SNVs were identified using **VarScan somatic**, where treated cells were compared with untreated counterparts. The following parameter modifications were made to minimize the occurrence of mutation calling artifacts: **-min-coverage 10**, **-min-var-freq 0.2**, **-somatic-p-value 0.05**, **-min-freq-for-hom 0.9**, **and -min-avg-qual 30**. The resulting SNVs were processed to identify high confidence mutation calls using **VarScan processSomatic** on default parameters, these high confidence calls were used to generate consensus mutation calls.

Strelka was executed with default parameters to compare tumor and normal samples, using the suitable reference genome. The SNV calls obtained were used for the identification of consensus mutations. Furthermore, the BAM files were used to generate a third set of SNVs using Somatic Sniper, with default parameters except for **-Q 40 -G -L**. The developers recommend a minimum somatic score of 40 for BWA aligned reads to avoid reporting false positives of loss-of-heterozygosity and gain-of-reference mutations in the final output. The resultant file was additionally used for the identification of consensus mutations.

To address potential artifacts associated with mutation calling and sequencing by different callers, we aggregated the consensus from three specific callers (Strelka, Somatic Sniper, and VarScan) for SNVs. The process was accomplished using a customized python script that necessitates the presence of mutations in all three files of the sample. The individual consensus mutation files were then combined, and any mutations found in more than 25% of samples from the same genotype were excluded due to a high probability of representing a preexisting variant compared with the reference genome or a sequencing/mutation calling artifact. The concatenated files were then divided into individual files, categorized by treatment, genotype, or both, as per the analysis requirements.

### Mutation spectra

Mutation spectra were constructed by combining single-nucleotide variants within subtelomeric regions, defined as the terminal 20 kb of each chromosome, and internal regions, comprising the remainder of the genome. Mutations were categorized by trinucleotide sequence context and pyrimidine-based mutations for internal and all types for the subtelomeric regions. To account for regional differences in nucleotide composition, mutation counts were normalized to mutations per megabase by dividing the number of mutations in each trinucleotide context by the genomic frequency of the corresponding context within the same region.

To estimate ssDNA-specific mutational processes in subtelomeric regions, we corrected for background dsDNA-associated mutation rates by subtracting mutation rates observed in internal genomic regions from those observed in subtelomeric regions. This approach accounts for the fact that subtelomeric DNA is transiently rendered single-stranded during a subset of resection events, estimated to occur ∼15% of the time. Accordingly, mutation rates measured in internal regions provide an empirical baseline for dsDNA mutagenesis that can be subtracted to isolate mutations attributable to ssDNA UV exposure.

### Chromosome arm loss analysis

Telomere loss was quantified from indexed BAM files using a custom Python workflow. For each sample, chromosome arms were defined from the reference FASTA index, and the terminal 20 kb of each chromosome end was divided into four 5-kb bins. Mean sequencing depth was calculated for each subtelomeric bin using **samtools bedcov** and normalized to the sample-specific internal chromosomal mean depth, defined as all genomic regions excluding the terminal 20 kb from each chromosome end. A subtelomeric bin was classified as lost if its mean normalized depth was <0.10 of the internal mean depth, and a chromosome arm was classified as lost if any bin within that arm met this criterion. Per-sample telomere-loss burden was summarized as the total number of lost chromosome arms and aggregated by genotype. Samples with low internal sequencing depth or outlier internal depth values, defined as >2 SDs from the genotype mean, were excluded from genotype-level plotting and summary statistics.
