# Supplementary material for: Widespread atypical UV-induced mutations form in single-stranded DNA
Source: Sci Adv. 2026 Sep 11;12(37):eaeg5184. doi: 10.1126/sciadv.aeg5184 (PMC13564860; doi:10.1126/sciadv.aeg5184)
Supplement: Supplementary file 1 [file sciadv.aeg5184_sm.pdf]

Science Advances
AAAS

Supplementary Materials for

Widespread atypical UV-induced mutations form in single-stranded DNA

Cameron Cordero *et al.*

Corresponding author: Steven A. Roberts, srober23@med.uvm.edu

Sci. Adv. **12**, eaeg5184 (2026)
DOI: 10.1126/sciadv.aeg5184

The PDF file includes:

Figs. S1 to S7
Legends for tables S1 to S6

Other Supplementary Material for this manuscript includes the following:

Tables S1 to S6

Figure S1

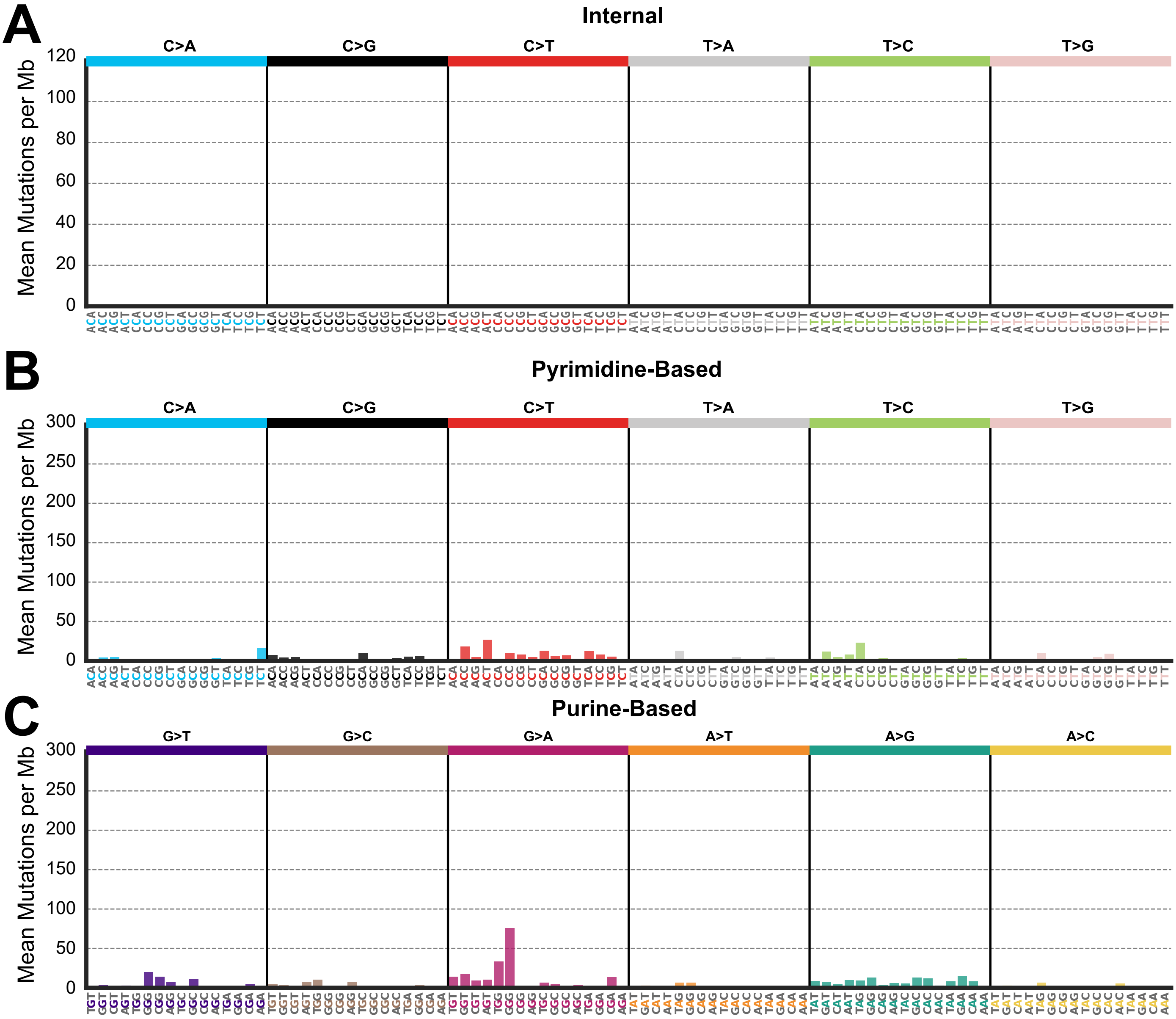
A
Internal
C>A
C>G
C>T
T>A
T>C
T>G
Mean Mutations per Mb
B
Pyrimidine-Based
C
Purine-Based
G>T
G>C
G>A
A>T
A>G
A>C

Figure S1. Mutation spectra from whole genome sequencing of untreated *cdc13-1* yeast. (a) Mutation profile for untreated *cdc13-1* yeast for mutations in the internal region, **(b)** pyrimidine originating mutations in the subtelomeric regions, and **(c)** purine originating mutations in the subtelomeric regions. Data are presented as mean values of the mutation frequencies for each substitution type (e.g., C>A, C>G, etc.) and trinucleotide context is depicted. The middle base of each trinucleotide context is mutated.

Figure S2

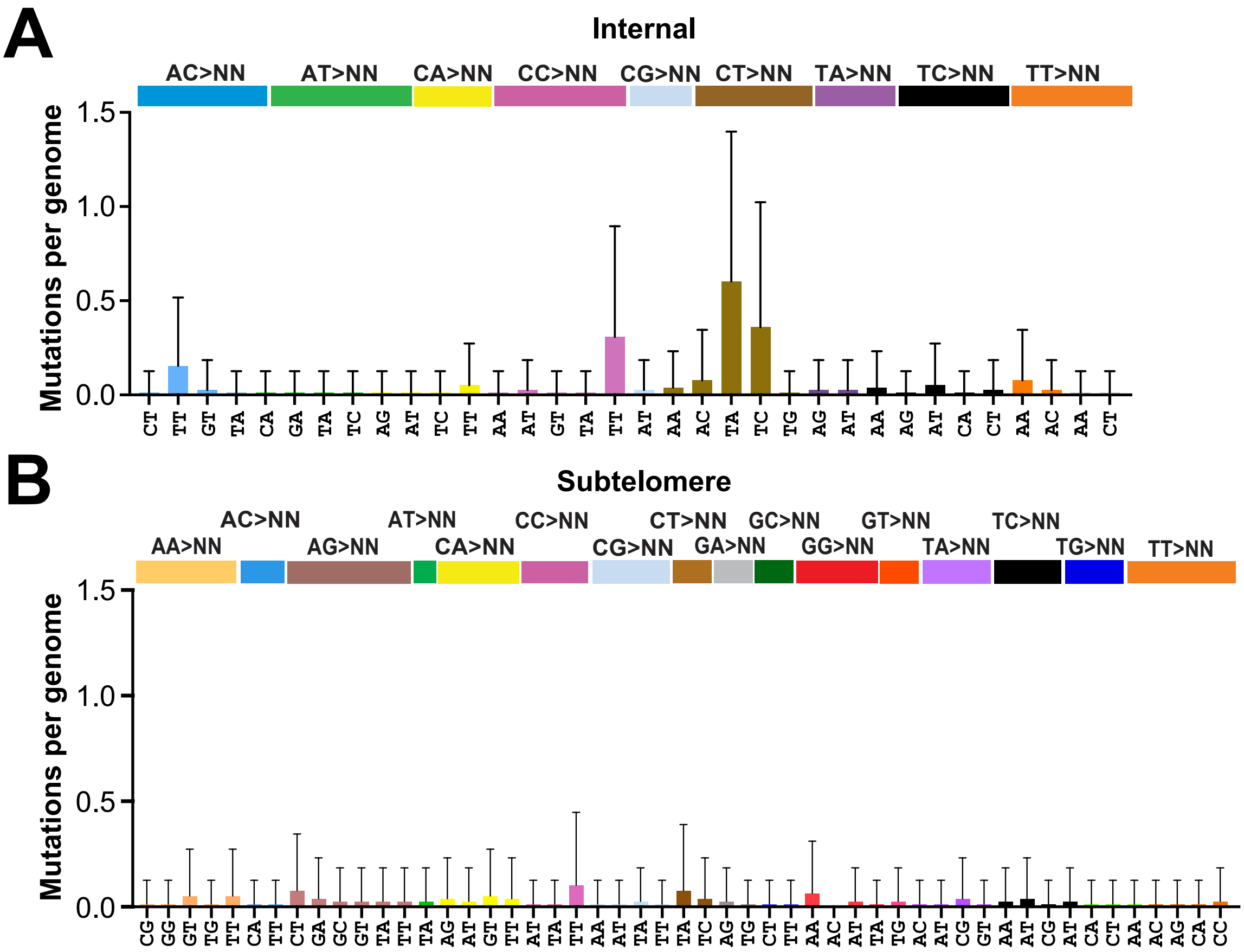
A
Internal
AC>NN
AT>NN
CA>NN
CC>NN
CG>NN
CT>NN
TA>NN
TC>NN
TT>NN
Mutations per genome
1.5
1.0
0.5
0.0
CT TT GT TA CA GA TA TC AG AT TC TT AA AT GT TA TT AT AA AC TA TC TG AG AT AA AG AT CA CT AA AC AA CT
B
Subtelomere
AA>NN
AC>NN
AG>NN
AT>NN
CA>NN
CC>NN
CG>NN
CT>NN
GA>NN
GC>NN
GG>NN
GT>NN
TA>NN
TC>NN
TG>NN
TT>NN
Mutations per genome
1.5
1.0
0.5
0.0
CG GG GT TG TT CA TT CT GA GC GT TA TT TA AG AT GT TT AT TA TT AA AT TA TT TA TC AG TG CT TT AA AC AT TA TG AC AT CG GT AA AT CG AT CA CT AA AC AG CA CC

Figure S2. Tandem substitution spectrum induced by UVB irradiation of *cdc13-1* yeast.
The frequency per genome of tandem substitutions in **(a)** internal regions and **(b)** subtelomeric regions of serially end-resected and irradiated *cdc13*-1 yeast.

Figure S3

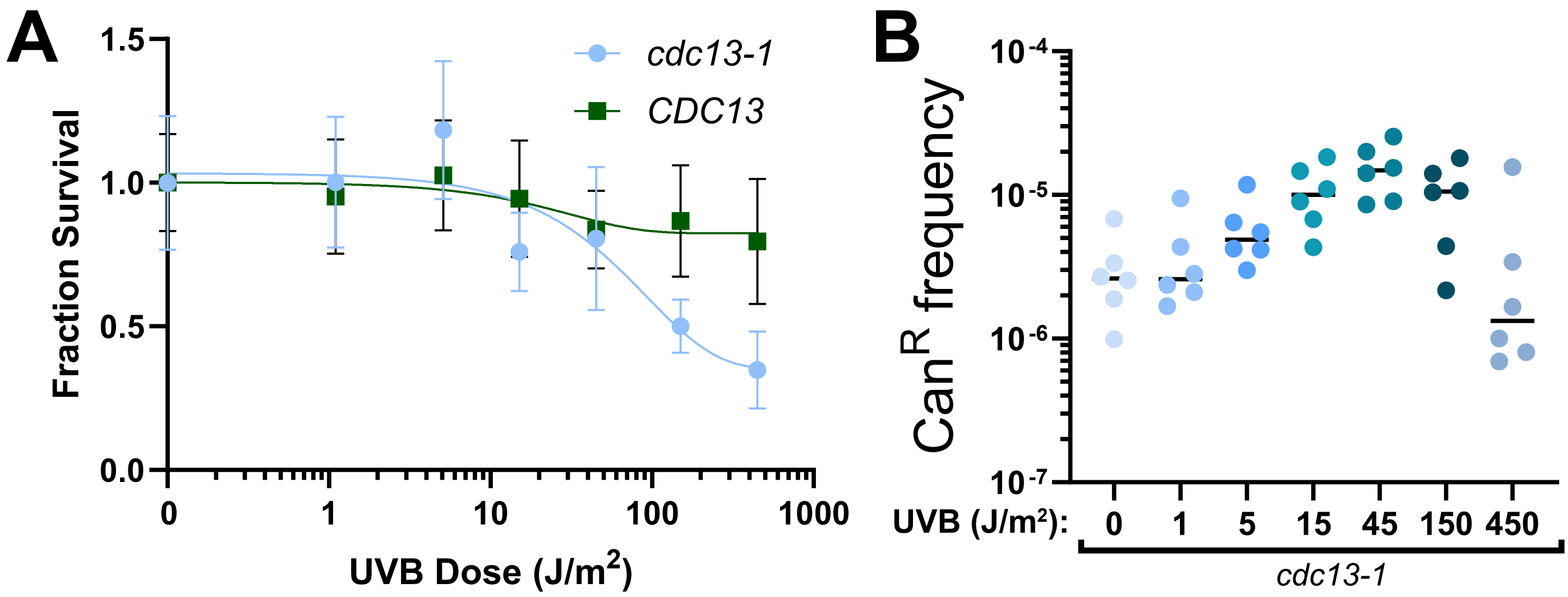

A
1.5
1.0
0.5
0.0
Fraction Survival
cdc13-1
CDC13
0
1
10
100
1000
UVB Dose (J/m2)
B
10-4
10-5
10-6
10-7
CanR frequency
UVB (J/m2):
0
1
5
15
45
150
450
cdc13-1

Figure S3. UVB Dose response in ssDNA. (a) Survival and **(b)** CAN-R of *cdc13*-1 and *CDC13* yeast following incubation at 37°C for 6 hours and exposure to increasing doses of UVB light.

Figure S4

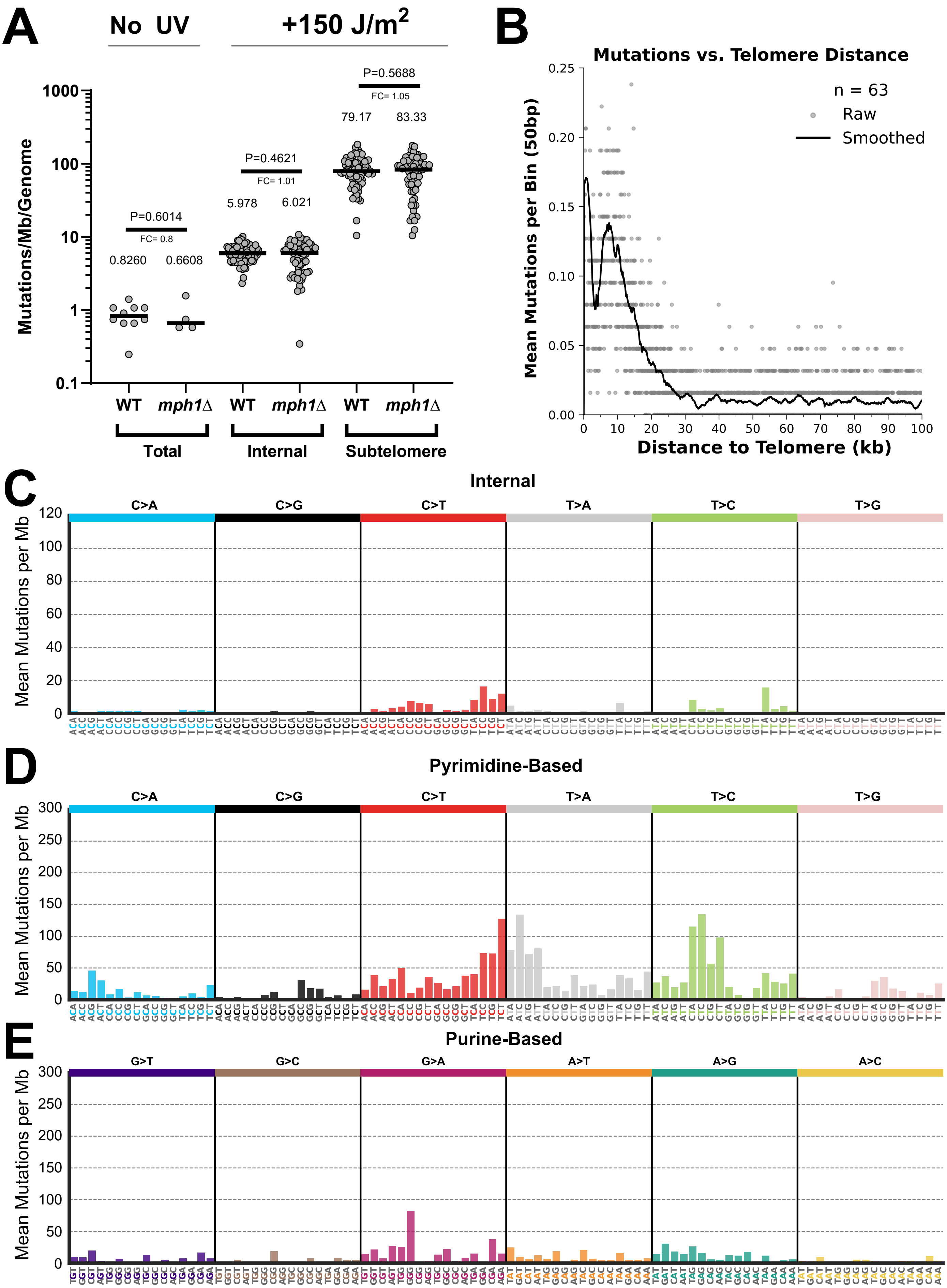
A
No UV
+150 J/m2
Mutations/Mb/Genome
1000
100
10
1
0.1
P=0.6014
FC= 0.8
0.8260
0.6608
P=0.4621
FC= 1.01
5.978
6.021
P=0.5688
FC= 1.05
79.17
83.33
WT
mph1Δ
WT
mph1Δ
WT
mph1Δ
Total
Internal
Subtelomere
B
Mutations vs. Telomere Distance
Mean Mutations per Bin (50bp)
n = 63
Raw
Smoothed
0.25
0.20
0.15
0.10
0.05
0.00
0
10
20
30
40
50
60
70
80
90
100
Distance to Telomere (kb)
C
Internal
C>A
C>G
C>T
T>A
T>C
T>G
Mean Mutations per Mb
120
100
80
60
40
20
0
D
Pyrimidine-Based
C>A
C>G
C>T
T>A
T>C
T>G
Mean Mutations per Mb
300
250
200
150
100
50
0
E
Purine-Based
G>T
G>C
G>A
A>T
A>G
A>C
Mean Mutations per Mb
300
250
200
150
100
50
0

Figure S4. Mutation spectra from whole genome sequencing of *cdc13-1 mph1*Δ UVB irradiated yeast. (a) Mutations per Mb in un-exposed and UVB exposed (150 J/m^2) *cdc13-1 mph1*Δ yeast (exposed 15x) in the internal and subtelomeric regions. The data was compared by a two-sided Mann–Whitney rank sum test. Median values are shown above the data, p-value and fold change (FC) listed above and below the statistical comparison bracket respectively. **(b)** The number of mutations occurring per 50 bp across the entire genome per sample in UVB exposed *cdc13-1 mph1*Δ yeast (n=63). **(c)** Mutation profile for UVB exposed (150 J/m^2) *cdc13-1 mph1*Δ yeast for mutations in the internal region, **(d)** pyrimidine originating mutations in the subtelomeric regions, and **(e)** purine originating mutations in the subtelomeric regions. Data are presented as mean values of the mutation frequencies for each substitution type (e.g., C>A, C>G, etc.) and trinucleotide context is depicted. The middle base of each trinucleotide context is mutated.

Figure S5

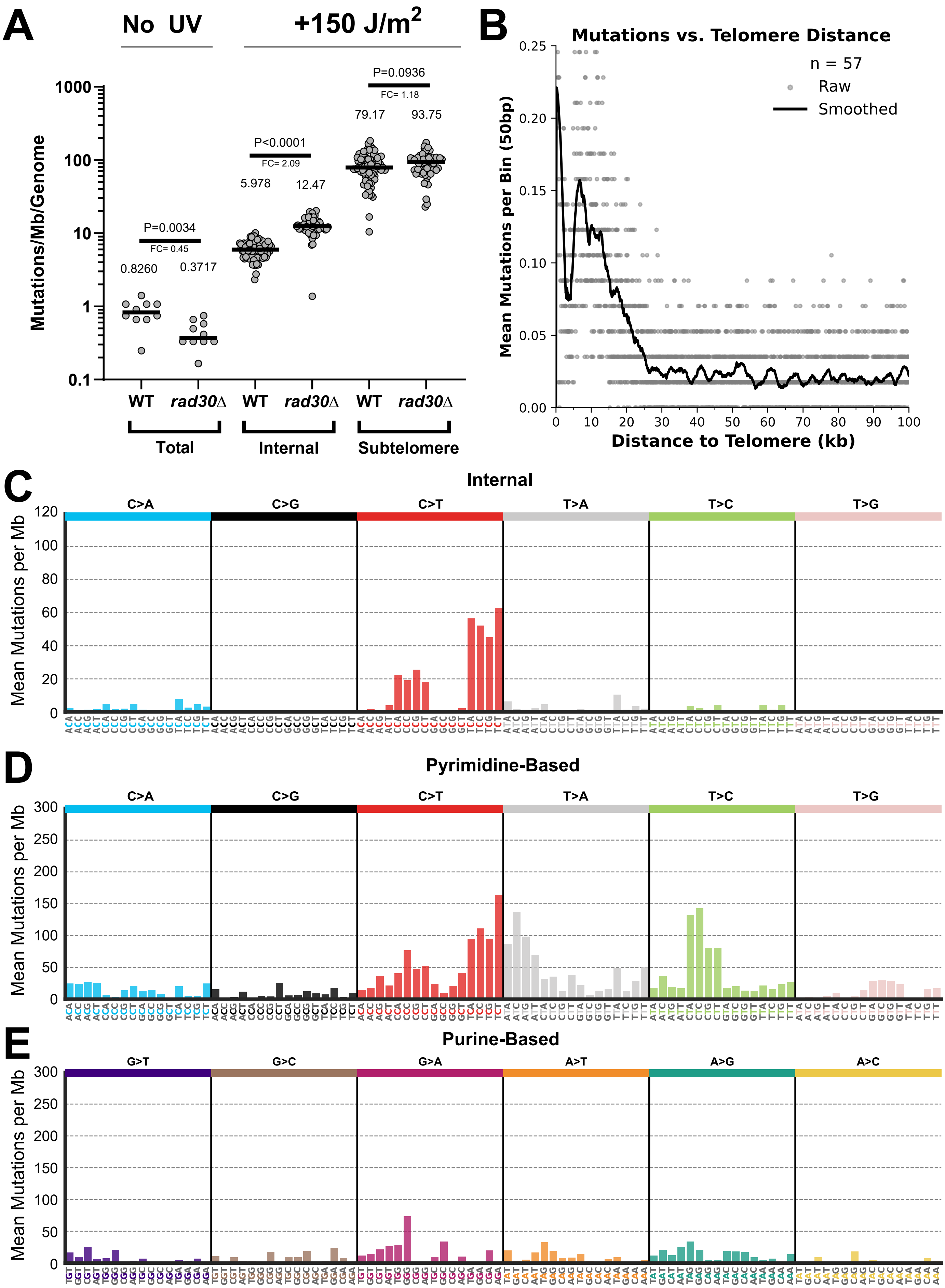
A
No UV
+150 J/m2
Mutations/Mb/Genome
1000
100
10
1
0.1
P=0.0034
FC= 0.45
0.8260
0.3717
P<0.0001
FC= 2.09
5.978
12.47
P=0.0936
FC= 1.18
79.17
93.75
WT
rad30Δ
Total
Internal
Subtelomere
B
Mutations vs. Telomere Distance
n = 57
Raw
Smoothed
Mean Mutations per Bin (50bp)
Distance to Telomere (kb)
C
Internal
C>A
C>G
C>T
T>A
T>C
T>G
Mean Mutations per Bin
D
Pyrimidine-Based
Mean Mutations per Mb
E
Purine-Based
G>T
G>C
G>A
A>T
A>G
A>C
Mean Mutations per Mb

Figure S5. Mutation spectra from whole genome sequencing of *cdc13-1 rad30*Δ UVB irradiated yeast. (a) Mutations per Mb in un-exposed and UVB exposed (150 J/m^2) *cdc13-1 rad30*Δ yeast (exposed 15x) in the internal and subtelomeric regions. The data was compared by a two-sided Mann–Whitney rank sum test. Median values are shown above the data, p-value and fold change (FC) listed above and below the statistical comparison bracket respectively. **(b)** The number of mutations occurring per 50 bp across the entire genome per sample in UVB exposed *cdc13-1 rad30*Δ yeast (n=57). **(c)** Mutation profile for UVB exposed (150 J/m^2) *cdc13-1 rad30Δ* yeast for mutations in the internal region, **(d)** pyrimidine originating mutations in the subtelomeric regions, and **(e)** purine originating mutations in the subtelomeric regions. Data are presented as mean values of the mutation frequencies for each substitution type (e.g., C>A, C>G, etc.) and trinucleotide context is depicted. The middle base of each trinucleotide context is mutated.

Figure S6

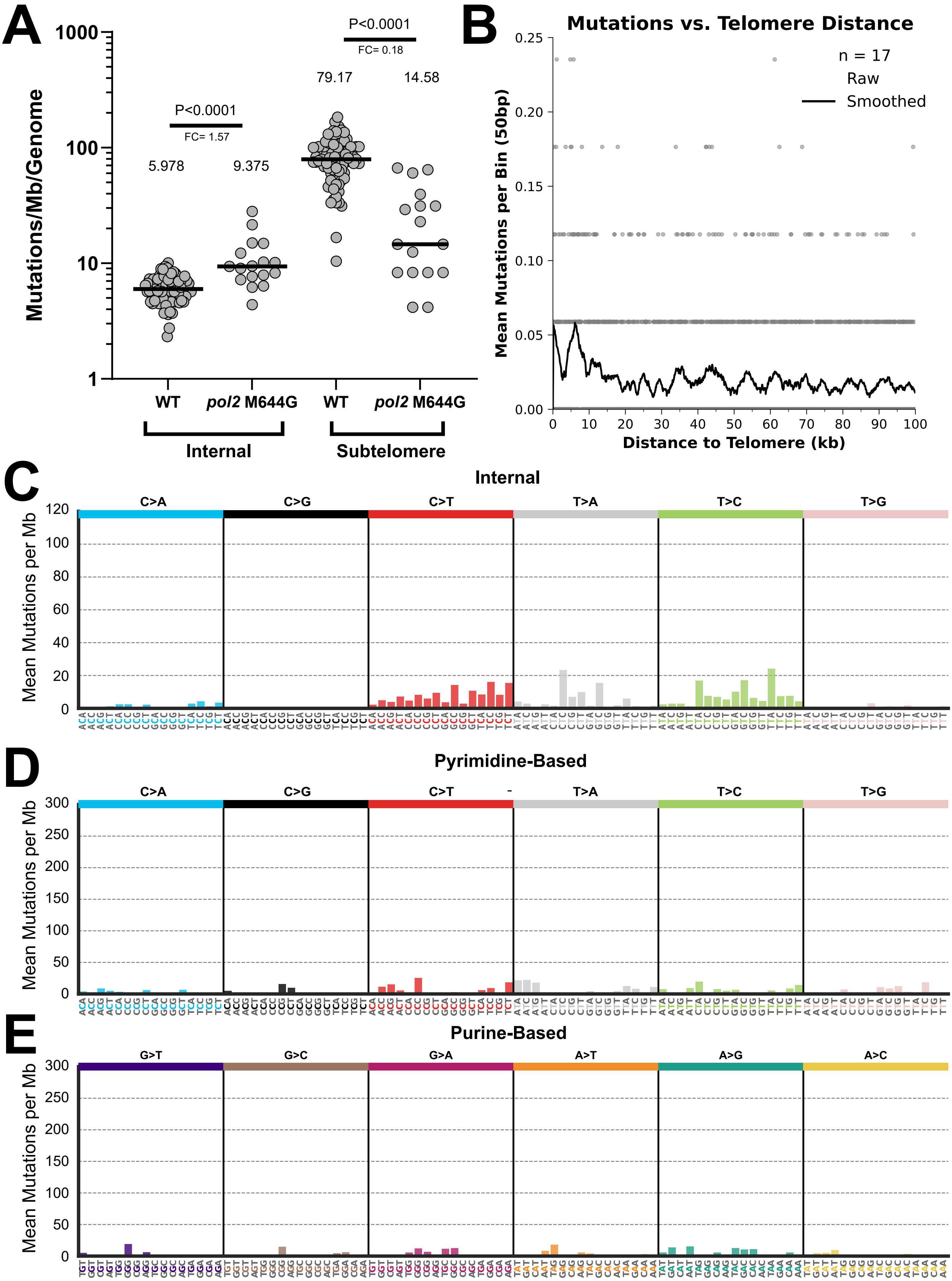
A
B
Mutations vs. Telomere Distance
n = 17
Raw
Smoothed
Mean Mutations per Bin (50bp)
Distance to Telomere (kb)
Mutations/Mb/Genome
P<0.0001
FC= 1.57
P<0.0001
FC= 0.18
5.978
9.375
79.17
14.58
WT
pol2 M644G
WT
pol2 M644G
Internal
Subtelomere
C
Internal
D
Pyrimidine-Based
E
Purine-Based
Mean Mutations per Mb
C>A
C>G
C>T
T>A
T>C
T>G
G>T
G>C
G>A
A>T
A>G
A>C

Figure S6. Mutation spectra from whole genome sequencing of *cdc13-1 pol2* M644G UVB irradiated yeast. (a) Mutations per Mb in UVB exposed (150 J/m^2) *cdc13-1 pol2* M644G yeast (exposed 15x) in the internal and subtelomeric regions. The data was compared by a two-sided Mann–Whitney rank sum test. Median values are shown above the data, p-value and fold change (FC) listed above and below the statistical comparison bracket respectively. **(b)** The number of mutations occurring per 50 bp across the entire genome per sample in UVB exposed *cdc13-1 pol2* M644G yeast (n=17). **(c)** Mutation profile for UVB exposed (150 J/m^2) *cdc13-1 pol2* M644G yeast for mutations in the internal region, **(d)** pyrimidine originating mutations in the subtelomeric regions, and **(e)** purine originating mutations in the subtelomeric regions. Data are presented as mean values of the mutation frequencies for each substitution type (e.g., C>A, C>G, etc.) and trinucleotide context is depicted. The middle base of each trinucleotide context is mutated.

Figure S7

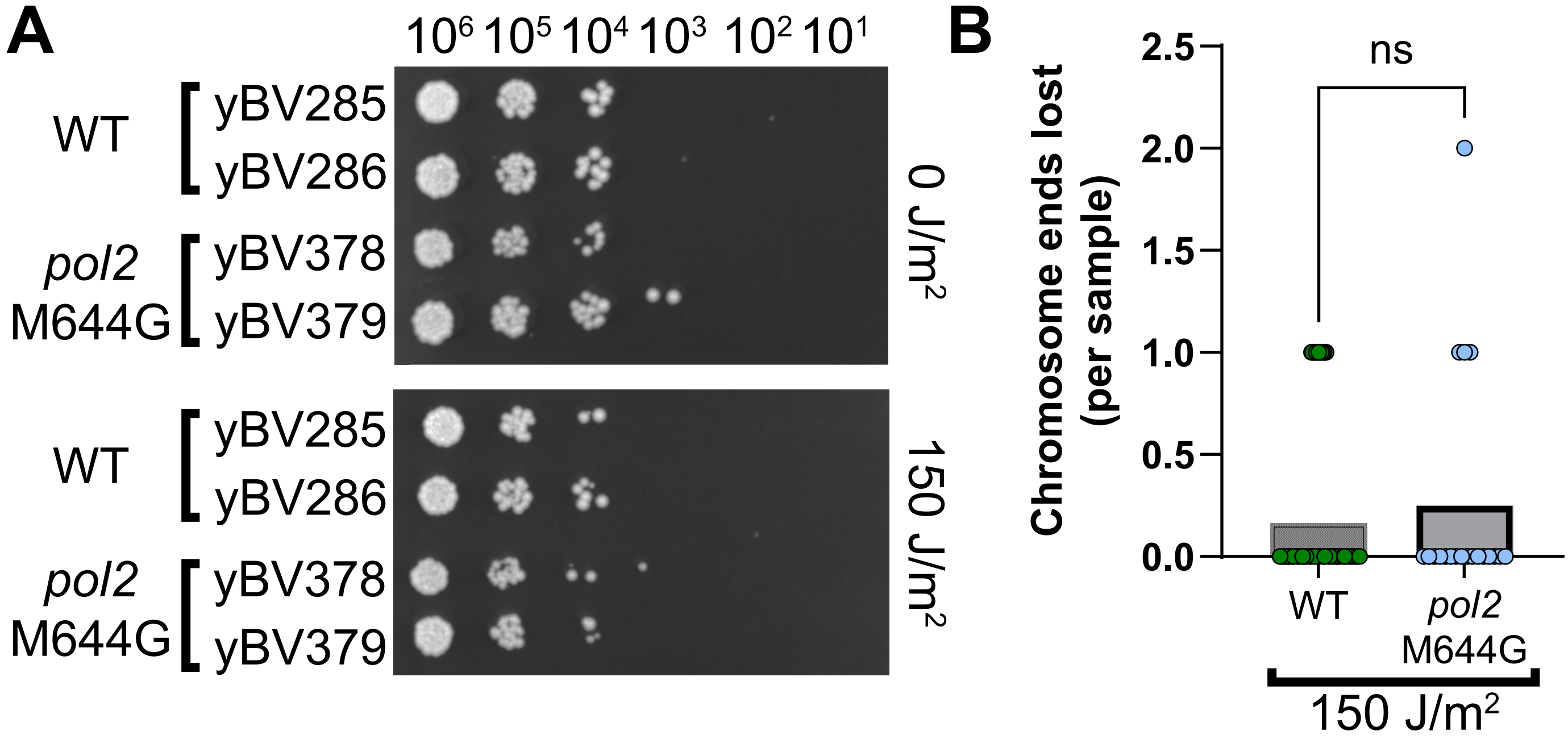

A
10^6 10^5 10^4 10^3 10^2 10^1
WT
yBV285
yBV286
pol2 M644G
yBV378
yBV379
0 J/m2
WT
yBV285
yBV286
pol2 M644G
yBV378
yBV379
150 J/m2
B
Chromosome ends lost (per sample)
2.5
2.0
1.5
1.0
0.5
0.0
ns
WT
pol2 M644G
150 J/m2

Figure S7. Growth of *cdc13-1* WT and *pol2* M644G UVB irradiated yeast. (a) Ten-fold serial dilutions of WT and *pol2* M644G yeast were spotted at YPDA media, temperature-shifted to 37°C for 6 hours, exposed to 0 or 150 J/m^{2} UVB, and growth observed over the course of 4 days at 23°C. (b) Number of subtelomeric deletions per genome in UV-irradiated WT (green) and *pol2* M644G (blue) yeast. ns = not significant. Chromosome ends were considered deleted if the mean sequencing depth within a 5 kb region of the subtelomere was less than 10% of the mean internal sequencing depth of the sample.

Table S1: List of Mutations identified in yeast whole genome sequencing

Table S2: Yeast Strains

Table S3: Statistical comparisons evaluating UV-induction and photolyase resistance of for mutations in UVB-irradiated yeast by χ^2.

Table S4: Statistical comparisons of photoreactivation of UV-induced mutations using χ^2.

Table S5: Statistical comparison of internal region UV-induced mutations between WT, *mph1*Δ, *rad30*Δ, and *pol2* M644G yeast using χ^2.

Table S6: Statistical comparison of subtelomere region UV-induced mutations between WT, *mph1*Δ, *rad30*Δ, and *pol2* M644G yeast using χ^2.
